# Anti-aging efficacy of solid-state fermented ginseng with *Aspergillus cristatus* and its active metabolites

**DOI:** 10.3389/fmolb.2022.984307

**Published:** 2022-09-29

**Authors:** Sang Cheol Park, Yura Ji, Jeoungjin Ryu, Seoyeon Kyung, Minji Kim, Seunghyun Kang, Young Pyo Jang

**Affiliations:** ^1^ Department of Life and Nanopharmaceutical Sciences, Graduate School, Kyung Hee University, Seoul, South Korea; ^2^ COSMAX BTI R&I Center, Bio Material Research Team, Seongnam-si, South Korea; ^3^ Department of Oriental Pharmaceutical Science, College of Pharmacy, Kyung Hee University, Seoul, South Korea; ^4^ Department of Biomedical and Pharmaceutical Sciences, Graduate School, Kyung Hee University, Seoul, South Korea

**Keywords:** fermented ginseng, golden flower fungus, *Aspergillus cristatus*, anti-aging, isodihydroauroglaucin, flavoglaucin, cosmeceutical

## Abstract

*Aspergillus cristatus* is a beneficial fungus of microbial fermented teas such as China’s Fuzhuan brick tea and Pu-erh tea, and is commonly called golden flower fungus (GFF) because its cleistothecium has a yellow millet or sand grain shape. Since natural materials fermented with GFF exhibit various physiological activities, a new active cosmeceutical ingredient was developed by solid-state fermentation of ginseng, a famous active material for healthy skin, with GFF. The extract of solid-state fermented ginseng with GFF (GFFG) exhibited potent anti-aging efficacy on the skin such as the increase of hyaluronic acid synthesis, aquaporin expression, and mRNA level of filaggrin in HaCaT keratinocyte. GFFG also inhibited the expression of MMP-1 increased by TNF-α in human dermal fibroblast. Sophisticated chromatographic and spectroscopic studies have elucidated isodihydroauroglaucin and flavoglaucin as the metabolites which were not present in ginseng extract nor GFF extract alone. Bioassay of these metabolites revealed that these compounds were part of active principles of GFFG. These results suggest that GFFG would be a potential active ingredient in anti-aging cosmeceutical products.

## Introduction

Biotransformation of herbal materials using beneficial fungi has been actively used to diversify active ingredients (Wu et al., 2021), enhance bioactivity (Tian et al., 2021), and reduce toxicity (Pinela et al., 2020). Among the beneficial fungi involved in the biotransformation of natural resources, *Aspergillus cristatus* is known to be the dominant fungus in the fermentation of Chinese Fuzhuan brick tea (Ge et al., 2016). Called “Golden Flower Fungus” because of its yellow cleistothecium color, this dominant taxon plays a key role in the production of Fuzhuan brick tea produced under controlled temperature and humidity conditions. *Aspergillus cristatus* is a beneficial fungus appearing in microbial fermentation tea in China and has been reported to play a variety of positive roles in the biotransformation of natural resources (Chen et al., 2021).

Natural materials fermented with golden flower fungus (GFF) are known to exhibit various physiological activities such as antioxidant activities in the case of rice Koji (Lee et al., 2021a) and anti-aging activity of *Panax notoginseng* (Lee et al., 2021b), so the fermentation using this fungus can be an effective way to develop new bio-active materials. Previous phytochemical studies reported that anthraquinones such as endocrocin derivatives and polyketides such as pannorin and flavoglaucin derivatives were major metabolites produced through fermentation of GFF (Miyake et al., 2009; Wu et al., 2021).

As a part of research on the development of new bioactive herbal materials that can be used in cosmeceutical products, this study investigated whether ginseng, a well-known herbal medicine used in various cosmeceutical products, has improved activity by GFF fermentation and to reveal and explore the activity of its active ingredients. In this paper, the metabolite changes of GFF fermented ginseng (GFFG) and general ginseng were analyzed and the anti-aging efficacy was evaluated through various bioassays of skin moisturizing, keratinocyte differentiation, and MMP-1 production, etc. Through sophisticated chromatographic and spectrometric studies, the active ingredients of solid fermented ginseng with GFF (GFFG) were also elucidated and their biological significances were evaluated.

This report aims to suggest that the GFF-fermented ginseng has great potential as a new natural ingredient for the cosmeceutical products, evidenced through the extensive biological and metabolomics studies.

## Materials and methods

### Chemicals and reagents

Dulbecco’s modified Eagle’s medium/F12 nutrient mixture Ham (DMEM/F12 3:1), antibiotic-antimycotic solution, trypsin-EDTA, phosphate buffered saline, Hank’s Balanced Salt Solution (HBSS) and fetal bovine serum (FBS) were purchased from Gibco (Grand Istand, NY, United States). Phosphoramidon disodium salt (Phosphoramidon), (+) sodium L-ascorbate (L-Ascorbate), epigallocatechin gallate (EGCG), N-succinyl-tri-alanyl-p-nitroanilide (STANA), albumin from bovine serum (BSA), Bradford reagent and trifluoroacetic acid (HPLC grade) were obtained from Sigma-Aldrich (St. Louis, MO, United States). Dimethyl sulfoxide (DMSO) was purchased from Amresco (Solon, OH, United States). Triton X-100 was obtained from Yakuri Pure Chemicals (Tokyo, Japan). Ethanol (HPLC grade) was obtained from Duksan Chem. Co. (Seoul, South Korea). Acetonitrile (HPLC grade) was purchased from Fisher Scientific Korea (Seoul, South Korea). Procollagen type I peptide (PIP) ELISA kit was obtained from Takara (Tokyo, Japan). Matrix Metalloproteinase-1 (MMP-1) human ELISA kit was purchased from Abcam (Cambridge, MA, United States). YOKUDELNA calibration kit was obtained from JEOL Ltd. (Tokyo, Japan).

### Preparation of solid state fermented ginseng with GFF (GFFG)

The fungal culture used in this study (*Aspergillus cristatus* Cosmax-GF) was obtained from Cosmax BTI R&I center (Seongnam, South Korea). *Aspergillus cristatus* was maintained on malt extract agar (malt extract, 20 g; glucose, 20 g; peptone, 1 g; agar, 20 g) at 28°C. Before the solid state fermentation step, raw ginseng roots (300 g) were washed with deionized water, sliced at 2 cm intervals, and autoclaved at 121°C for 60 min. *Aspergillus cristatus* was incubated on malt extract agar media at 28°C and the spore was collected by the treatment of 0.01% tween-20 using Neubauer chamber. The amount of final fungal spore was adjusted to approximately 2.0 × 10^5^ cfu/ml. *Aspergillus cristatus* (2%, v/w) was directly inoculated in ginseng roots for the solid state fermentation. After inoculation, the samples were incubated for 4 days at 28 °C with 85% humidity. Solid state fermented ginseng was dried until they reached below 12% humidity at 60°C and then pulverized. Seventy percentile ethanol extract of this pulverized GFFG was used in biological evaluation and metabolomics studies.

### Cell culture and *in vitro* assay

Human dermal fibroblast cells (HDFs) were obtained from American Type Culture Collection (ATCC; Manassas, VA, United States). HDFs are primary cells isolated from adult skin without detection of *mycoplasma*, hepatitis B virus, hepatitis C virus, HIV-1, bacteria, yeast, and other fungi. The HDFs were cultured in DMEM/F12 3: 1 mixed medium with 1% antibiotic-antimycotic (v/v) and 10% FBS (v/v) at 37 °C in an incubator (HERAcell 150i, Thermo Scientific, Waltham, MA, United States) containing 5% CO_2_.

The immortalized human keratinocytes (HaCaT) and human fibroblasts (Hs68) were obtained from ATCC and cultured in Dulbecco’s modified Eagle’s medium (DMEM; Hyclone Laboratories, Inc., Logan, UT, United States) supplemented with 1% antibiotic-antimycotic solution and 10% fetal bovine serum in an atmosphere of 5% CO_2_ at 37°C. HaCaT were maintained until 80% confluence and then cells were treated with each sample of ginseng, *A. cristatus* lysate, and GFFG (100 μg/ml) in serum-free medium and incubated for 24 h. Retinoic acid (RA; 1 μM) was used as a positive control for the hydration bioassay. Hs68 were also cultured in serum-free medium for 24 h and then exposed or not exposed to UVB light (20 mJ/cm^2^) with UVB light source. Then cells were treated with isodihydroauroglaucin (gh1) and flavoglaucin (gh4) samples of indicated concentration (5–500 ng/ml) in serum-free medium and incubated for 24 h. EGCG (1 μM) was used as a positive control for the anti-aging bioassay.

### Cell viability

Cell viability was determined using the 3-(4,5-dimethylthiazol-2-yl)-2,5-diphenyltetrazolium bromide (MTT; Sigma-Aldrich, St. Louis, MO, United States) colorimetric assay. HaCaT cells were cultured overnight in 96-well plates (5 × 10^4^ cells/well). Cells were treated with various concentrations of GFFG and incubated in serum-free medium for 24 h. The cell medium was replaced with 50 μL MTT solution (0.5 mg/ml) and incubated for an additional 4 h. After washing the cells, the insoluble formazan products were dissolved in 200 μL dimethyl sulfoxide. Absorbance at 550 nm was determined by spectrophotometry using a VersaMax tunable microplate reader (Molecular Devices Inc., Sunnyvale, CA, United States).

### Real-time RT-PCR

The total RNA from keratinocytes and fibroblasts was isolated using RNAiso reagent according to recommended protocols. cDNA was synthesized from 1 μg of total RNA using a reverse transcription premix (Elpis-biotech, Daejeon, Korea) for 45 min at 45°C and 5 min at 95°C. Gene expression signals were quantified by real-time polymerase chain reaction (PCR) amplification and data were analyzed using StepOne Plus software (Applied Biosystems, Foster City, CA, United States). Real-time PCR amplification reactions were performed using an SYBR Green PCR Master Mix with premixed ROX (Applied Biosystems, Foster City, CA, United States). The following primer pairs (Bioneer, Daejeon, Korea) were used in the reactions performed in an ABI 7300 following the manufacturer’s protocol: β-actin (F: 5′-GGC CAT CTC TTG CTC GAA GT-3′ and R: 5′-GAC ACC TTC AAC ACC CCA GC-3′), FLG (F: 5′-AGT GCA CTC AGG GGG CTC ACA-3′ and R: 5′-CCG GCT TGG CCG TAA TGT GT-3′), HAS3 (F: 5′-CTT AAG GGT TGC TTG CTT GC-3′ and R: 5′-GTT CGT GGG AGA TGA AGG AA-3′), AQP3 (F: 5′-GTC ACT CTG GGC ATC CTC AT-3′ and R: 5′-CTA TTC CAG CAC CCA AGA AGG-3′), MMP-1 (F: 5′-CGA ATT TGC CGA CAG AGA TGA-3′ and R: 5′-GTC CCT GAA CAG CCC AGT ACT T′), COL1A1 (F: 5′-GAG​GGC​CAA​GAC​GAA​GAC​ATC-3′ and R: 5′- CAG​ATC​ACG​TCA​TCG​CAC​AAC-3′), and FBN1 (F: 5′- AAT​GTC​AGA​CGA​AGC​CAG​GG-3′, R: 5′- GAT​TTG​GTG​ACG​GGG​TTC​CT-3′). Real-time PCR was performed using an Applied Biosystems 7,300 real-time PCR system (Applied Biosystems, Waltham, MA, United States). The reaction conditions were as follows: initiation at 50 °C for 2 min and 95 °C for 10 min, followed by 40 cycles of 95 °C for 10 s and 60 °C for 1 min. Expression of β-actin was used as an internal control.

### Assay of MMP-1 secretion

Human dermal fibroblast cells (HDFs) were cultured in 24-well plates at a density of 5 × 10^4^ cells/well and incubated in complete culture medium for 24 h. The cells were then induced with TNF-α, treated with each sample (100 μg/ml), and incubated in serum-free medium for 24 h. Then, the supernatant was taken and collagenase inhibitory activity was measured at 450 nm using MMP-1 Human ELISA Kit. The content of MMP-1 was corrected for total protein content and compared to positive control, retinol (20 μg/ml).

### Extraction and isolation of metabolites of GFFG

The GFFG 70% ethanol extract (15 g) was prepared by freeze-drying the fermented ginseng (150 g) extract with 70% ethanol sonication. The extract was suspended in water and extracted 10 times with n-hexane to prepare an n-hexane fraction. The total n-hexane fraction was 1.3 g (yield; 8.7%) and aqueous fraction was 13.4 g (yield; 85.6%). In the hexane fraction, individual compounds were isolated using repeated HPLC separation. The hexane fraction dissolved in acetonitrile (100 mg/ml) was filtered through a 0.45-μm polytetrafluoroethylene (PTFE) filter, then was repeatedly injected to an Atlantis T3 C18 reverse-phase HPLC column (4.6 × 150 mm, 5 μm, Waters Corp., Milford, United States). The flow rate was 1.7 ml/min and the ultraviolet visible (UV/Vis) detection wavelength was set at 210 nm. The mobile phase was consisted of acetonitrile and distilled water and the gradient system was as follows: 69% (0–8.5 min), 69–100% (8.5–9 min), 100% (9–12 min), 100–69% (12–12.5 min) and 69% (12.5–15 min) (percent of acetonitrile). The peak 1 and 2 (gh-1 (10.2 mg) and gh4 (8.1 mg)) were collected at approximately 4.2 and 8.2 min, respectively. Their purity was confirmed to be greater than 95% by UPLC analysis.

### UPLC-PDA-ESI-TOF-MS analysis

Fifty milligram of each sample was dissolved in 1 ml 50% ethanol and filtered through a 0.22 µm PTFE filter (Whatman International Ltd, Maidstone, United Kingdom) for injection into the UPLC system. An UPLC analytical column (Acquity™ BEH C18 column, 1.7 µm, 2.1 × 50 mm, Waters Corp., Milford, United States) was used with Acquity™ BEH C18 VanGuard pre-column (1.7 µm, 2.1 × 5 mm) for Waters UPLC system (Acquity H-class, Waters Corp., Milford, MA, United States). A photodiode array (PDA) detector recorded between 210 and 400 nm. Chromatogram was monitored at 203 nm. The mobile phase was consisted of acetonitrile (solvent A) and distilled water with 0.1% trifluoroacetic acid (solvent B). Gradient conditions are 0–5 min, 5%–15%; 5–10 min, 15%–30%; 10–25 min, 30%–60%; 25–30 min, 60%–100% as percentage of solvent A. The column temperature was maintained at 25°C. The flow rate was 0.6 ml/min and the injection volume was 2.0 µL.

Molecular weight of detected compounds and calculated molecular formula were analyzed by TOF-MS (JMS-T100TD, JEOL Ltd., Tokyo, Japan) connected to an ESI source (JEOL Ltd., Tokyo, Japan) operated with Mass Center software (version 1.3.7). In the positive mode, the MS analysis conditions were set as follows: orifice 1 = 80 V, orifice 1 temperature = 80 °C; ring lens = 5 V; orifice 2 = 10 V; needle electrode = 2000 V; desolvating chamber temperature = 250°C; detector voltage = 2200 V, peak voltage = 1500 V; the flow rate of nitrogen gas used for nebulizing and desolvating was set to 1 and 3 L/min, respectively. Mass scale calibration was performed using a Yokudelna calibration kit (JEOL, Toyko, Japan). MS acquisition was set to a scan range of m/z 50–1,500.

### NMR study

The NMR spectra were recorded and measured using an Agilent DD2 700 MHz spectrometer (Santa Clara, CA, United States). Deuterated dimethylsulfoxide (DMSO-d6, Sigma-Aldrich) was used as NMR solvent and tetramethylsilane (Sigma-Aldrich) was used as an internal standard. Samples were placed in 5 mm NMR sample tubes (Sigma-Aldrich) and the analysis was performed at an elevated temperature of 25°C to improve spectral resolution.

### Statistical analysis

*In vitro* data are presented as mean ± standard deviation (SD) of at least 3 independent experiments. All data were analyzed by *t*-test. A p-value less than 0.05 was considered statistically significant.

## Results and discussion

### Cell viability of GFFG

**FIGURE 1:**
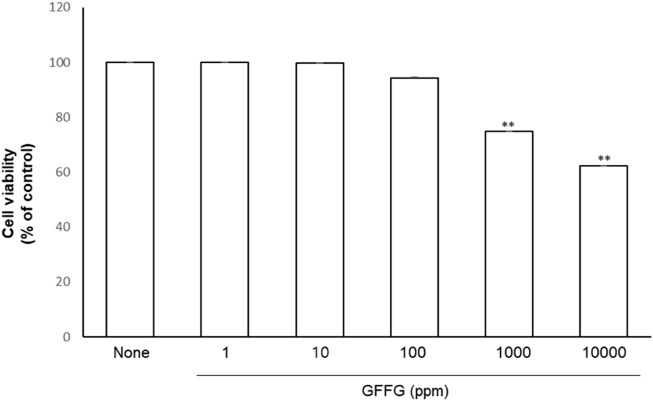
Cell viability evaluation of GFFG on HaCaT cells. **p < 0.01 compared with control group.

The effect of GFFG on cell viability was assessed by MTT assay. HaCaT cells were treated with GFFG (1–10000 μg/ml) and the viability was assessed after 24 h. The toxicity of GFFG increased at a concentration of 1,000 μg/ml on HaCaT cells (Figure 1). Therefore, the evaluation of anti-aging activity at 100 μg/ml concentration was studied.

### Skin moisturization *in vitro* test

**FIGURE 2:**
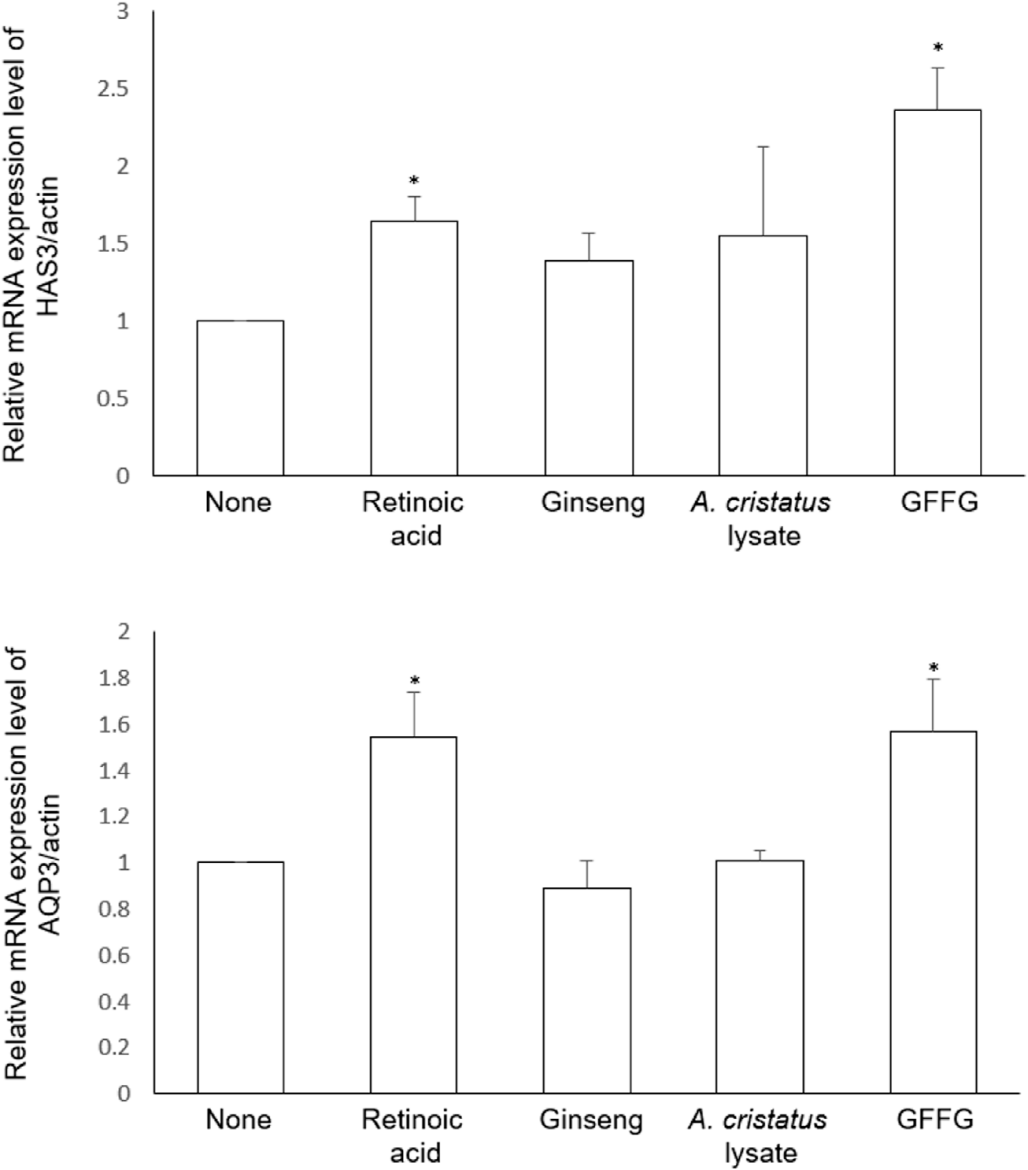
Efficacy on mRNA expression of HAS3 and AQP3 in keratinocytes treated with GFFG. Retinoic acid (1 µM) was compared as a positive control and the samples were evaluated for their activities at the same concentration of 100 μg/ml *p < 0.05 compared with control group.

With age, skin function deteriorates and skin moisture content decreases. In this study, we evaluated two biomarkers, HAS3 and AQP3, which play essential roles in skin hydration (Li et al., 2017; Nakamura et al., 2020). HAS3 is an enzyme involved in the synthesis of unbranched glycosaminoglycan hyaluronan or hyaluronic acid, which are the main components of the extracellular matrix. AQP3 is found in cell membranes and provides a pathway for water and glycerin. GFFG significantly increased HAS3 gene expression in keratinocytes, and its efficacy was stronger than that of retinoid acid (Figure 2; upper graph). The extract of ginseng and *A. cristatus* didn’t show significant increase in HAS3 gene. The data also showed that GFFG significantly increased AQP3 gene expressions in keratinocytes, (Figure 2; lower graph). Therefore, it is evaluated that GFFG activates two genes related to skin moisturizing and exhibits a skin moisturizing effect.

### Enhancement of skin barrier

**FIGURE 3:**
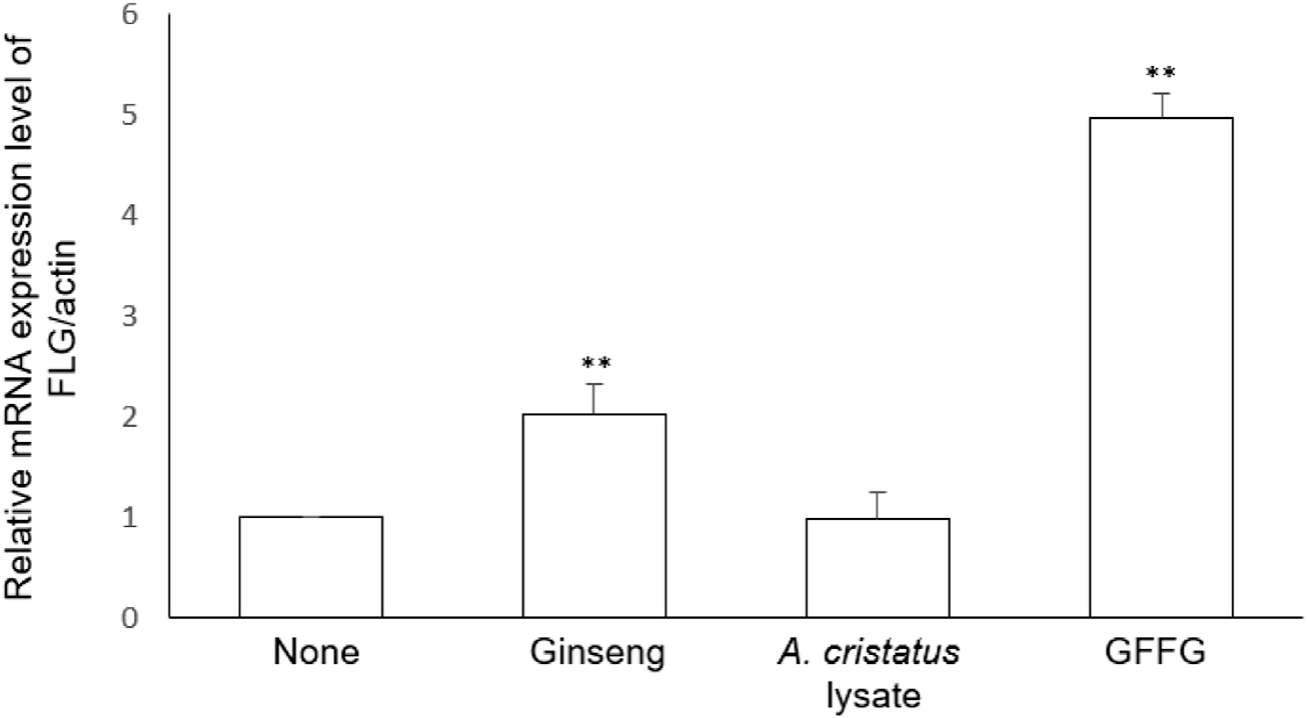
The mRNA expression level of filaggrin (FLG) in keratinocytes treated with GFsFG. The concentration of samples was 100 μg/ml **p < 0.01 compared with control group.

The cornified envelope is formed during keratinocyte differentiation and protects the skin from dehydration. By examining the mRNA level of filaggrin (FLG), a marker of keratinocyte differentiation, the ability of GFFG to enhance the formation of the cornified envelope was clarified. Filaggrin, which acts as one of the key factors for skin hydration, is produced by proteolysis of profilaggrin (Cepelak et al., 2019). When 100 μg/ml of GFFG was treated, the mRNA level of filaggrin was significantly increased compared to the untreated control group (*p* < 0.01) (Figure 3). From the results of skin moisturizing analysis including AQP3, HAS3, and FLG, it can be concluded that GFFG can protect the skin from the external environment by increasing the moisturizing efficacy of the skin.

### Inhibitory effects of GFFG on MMP-1 mRNA production

**FIGURE 4:**
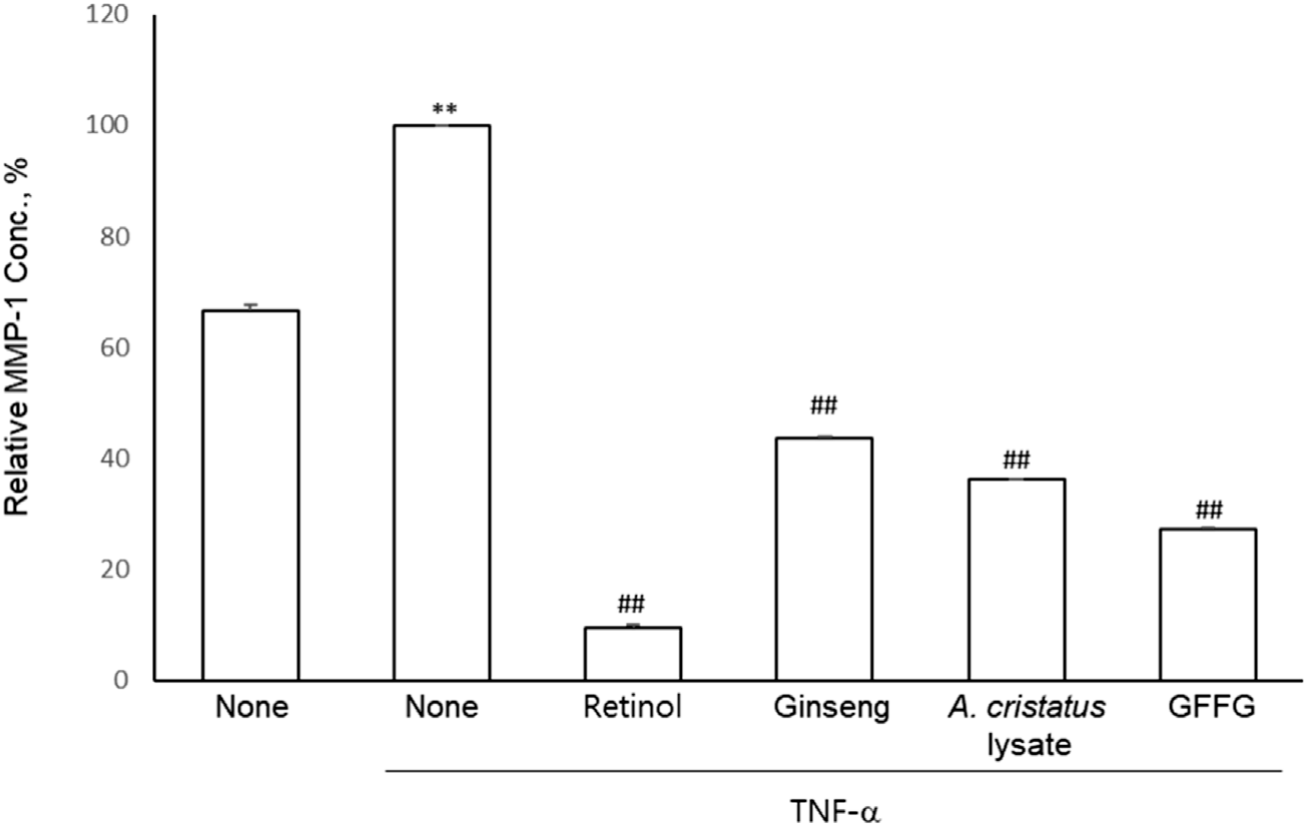
The inhibition of MMP-1 production in HDFs. Retinol (20 μg/ml) was compared as a positive control and the samples were evaluated at 100 μg/ml ^**^ *p* < 0.01 compared to blank group, ^##^ *p* < 0.01 compared to non-treated TNF-α group.

Matrix Metalloproteinases (MMPs) are another indicator of skin aging (Philips et al., 2007). To investigate the effect of GFFG on the expression or activity of collagenase, MMP-1 secretion was assessed by ELISA assay in dermal fibroblasts. TNF-α significantly increased MMP-1 secretion, and GFFG significantly inhibited the increase in MMP-1 secretion caused by TNF- α in fibroblasts (Figure 4). The results showed that GFFG can protect the extracellular matrix by inhibiting the production of MMP-1 and the degradation of collagen.

### Identification of active principles of GFFG

**FIGURE 5:**
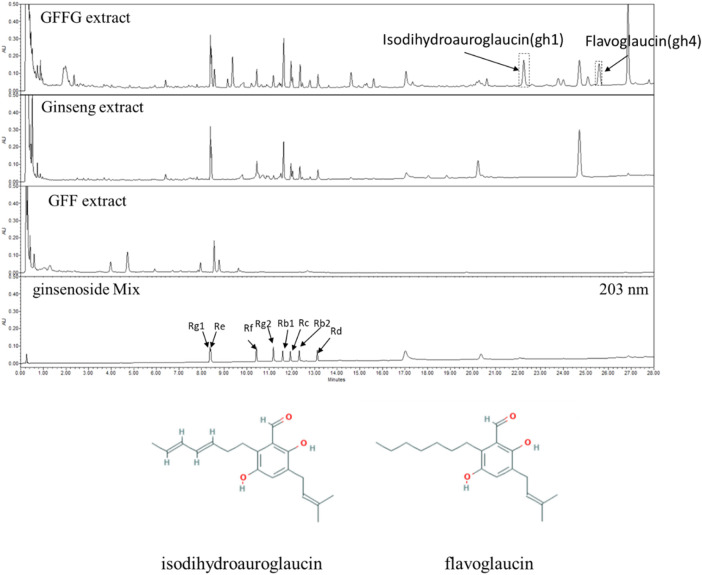
UHPLC analysis of GFF extract, ginseng extract and GFFG extract. Newly appeared gh1 (isodihydroauroglaucin) and gh4 (flavoglaucin) peaks were separated and their structures were represented at the below of chromatograms.

In order to identify the bioactive metabolites of GFFG, two compounds newly appeared in GFFG, which were not present in ginseng extract nor GFF extract, were isolated by the repeated HPLC separation (Figure 5). Detailed HPLC chromatogram with UV/Vis spectra of these peaks is represented in Supplementary Figure S1. By direct comparison of the NMR and MS data of isolated compounds with those reported in literature, the two compounds gh1 and gh4 were identified as isodihydroauroglaucin and flavoglaucin, respectively (Miyake et al., 2009). The MS spectra and NMR spectra were provided in Supplementary Figures S2, S3 and NMR data were summarized in Supplementary Table S1. The content of isodihydroauroglaucin was 0.098% and flavoglaucin was 0.071% in GFFG, respectively.

### Inhibitory effects of gh1 and gh4 on MMP-1 mRNA expression

**FIGURE 6:**
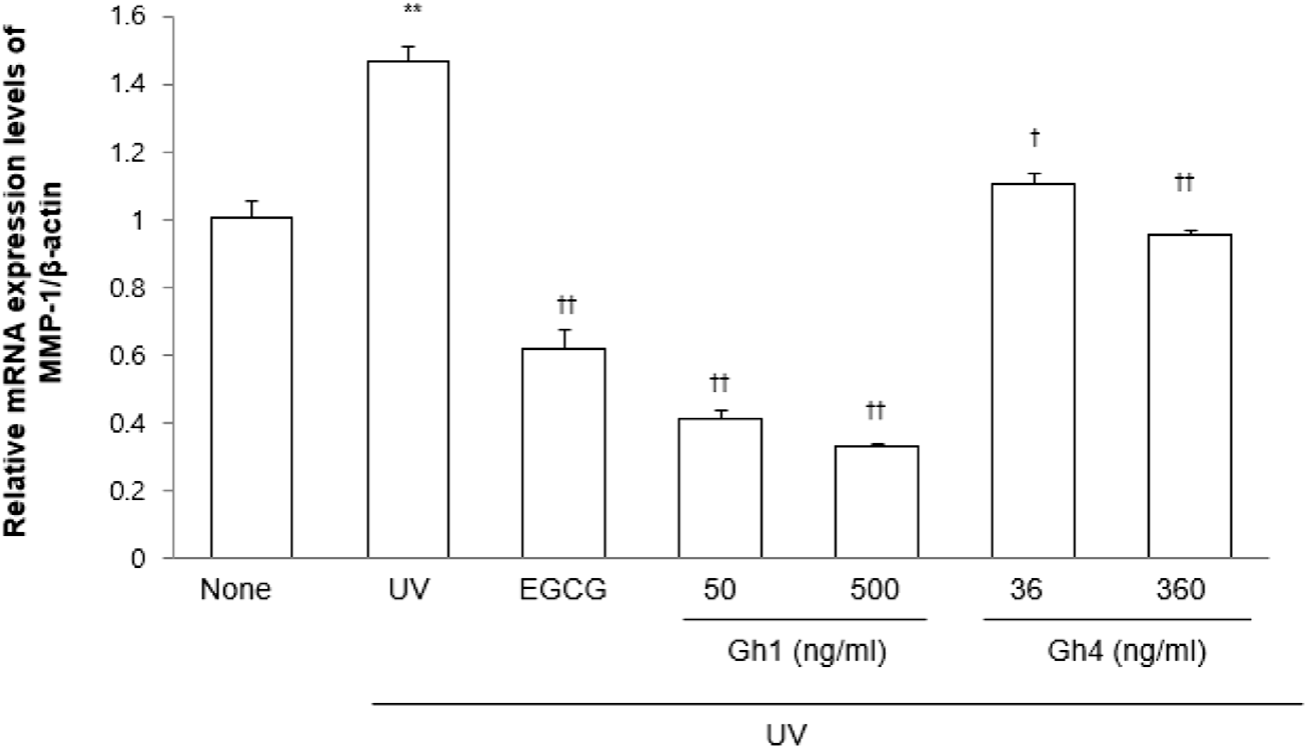
The inhibitory effects of GFFG on the MMP-1 mRNA expression. EGCG (1 μM) was used as a positive control. ^**^ *p* < 0.01 compared to blank group, ^†^ *p* < 0.05, ^††^ *p* < 0.01 compared to UVB treated group.

Because UVB irradiation induces collagenase MMP-1 expression and then stimulates wrinkle formation by degradation of dermal collagen (Dong et al., 2008; Wang, 2021), we evaluated the protection efficacy of gh1 and gh4 against UVB-induced MMP-1 mRNA expression in Hs68. UVB irradiation significantly induced MMP-1 mRNA expression compared to the UV-untreated group. However, gh1 and gh4 treatment dose-dependently decreased MMP-1 mRNA levels (Figure 6). This result suggests that gh1 and gh4 have an effect of protecting the skin from photoaging.

### Effects of gh1 and gh4 treatment on gene expressions of dermal extra cellular matrix (ECM) in Hs68 fibroblasts

**FIGURE 7:**
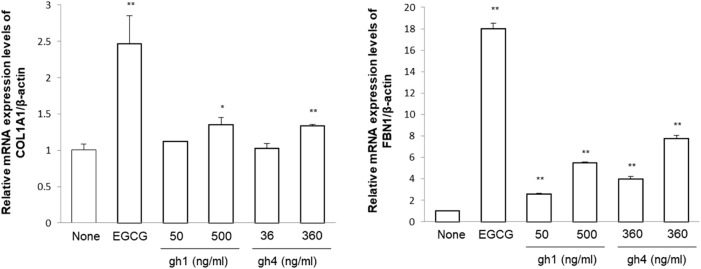
mRNA expression of COL1A1 and FBN1 in fibroblast. EGCG (1 μM) was used as a positive control. ^*^ *p* < 0.05, ^**^ *p* < 0.01 compared to blank group, repectively.

Dermal fibroblasts play a major role in generating ECM to maintain human skin structure and tissue regeneration. To evaluate the anti-aging effects of gh1 and gh4, ECM-related factor genes, COL1A1 and FBN1, were measured (Figure 7). Quantitative analysis of the mRNA expression level of COL1A1 showed a dose-dependent increase in the gh1 and gh4 treated group. In addition, FBN1 mRNA expression was also increased in the gh1 and gh4 treated groups compared to the untreated group. These results indicate that gh1 and gh4 exert anti-aging effects on the skin.

### Enhancement of HAS3 mRNA expression by the treatment of gh1 and gh4 in HaCaT keratinocytes

**FIGURE 8:**
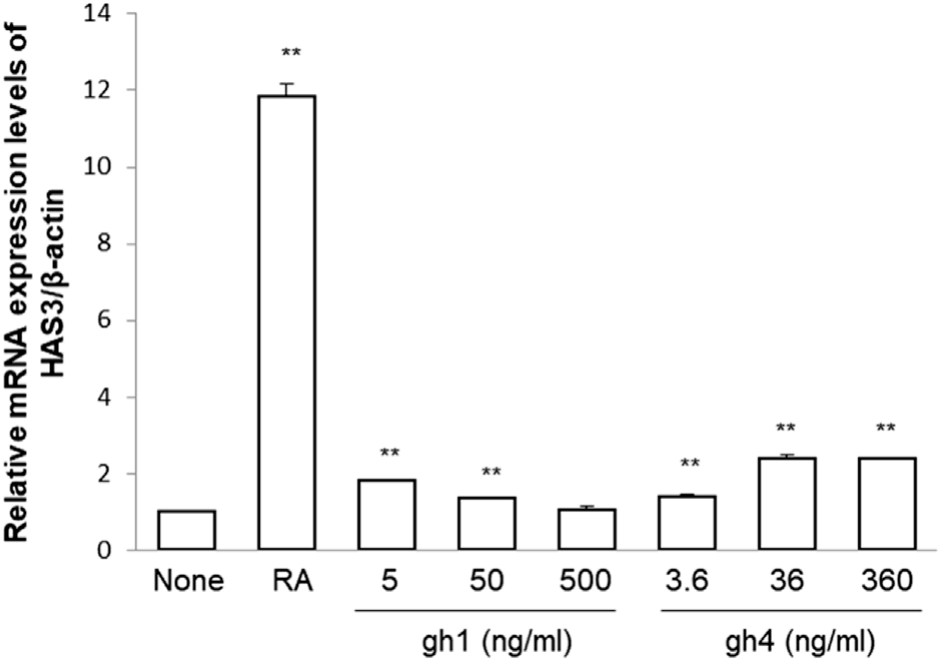
mRNA expression of HAS3 in keratinocytes treated with gh1 and gh4. Retinoic acid (1 μM) was used as positive control. ^**^ *p* < 0.01 compared to blank group.

The function of hyaluronic acid (HA) is known to prevent skin aging as well as water retention, so the effect of gh1 and gh4 on the mRNA expression of HAS3, an HA synthetase in keratinocytes, was reconfirmed. As shown in Figure 8, gh1 and gh4 increased the expression of the gene encoding HAS-3 in HaCaT cells. The efficacy of gh1 treatment was confirmed only at low concentrations, but gh4 treatment showed an upregulating effect of HAS3 in a dose-dependent manner. Although the efficacy was not as strong as that of retinoic acid, gh1 and gh4 can improve both skin moisturizing and skin aging by promoting the expression of HAS3 in HaCaT cells.

## Conclusion

Skin aging is mainly closely related to the decrease in moisture in the skin, and skin aging such as wrinkles and cracks in the skin often occurs in the elderly due to the decrease in skin moisture, which is caused by a decrease in the moisture content and barrier function of the skin. In order to develop effective natural materials that can prevent skin aging, new materials were sought through *in vitro* evaluation focusing on AQP3, HAS3, and MMP-1 mechanisms. Significant skin aging inhibitory activity was observed in GFFG solid-phase fermented using *Aspergillus cristatus*, a beneficial fungus best known as the dominant bacterium in fermentation of Chinese Fuzhuan brick tea. Two metabolites prominent in the GFFG extract were separated through chromatographic analysis, and their structures were identified by spectroscopic analysis. The two separated compounds were found to be substances with effective anti-aging activity through various activity evaluations related to skin aging. Although these compounds may not represent all the activities exhibited by GFFG extract, they may be used as effective quality control marker in future product development. It is not difficult to observe the case of increased efficacy or new activity through fermentation using beneficial microorganisms in natural raw materials. Therefore, efforts are being made to develop better biomaterials through the fermentation process for various herbal materials. However, one of the difficulties in product development through fermentation will be the development of a material with a consistent active ingredients profile. For this, a standardized manufacturing process is most important, but the discovery of quality markers that can effectively evaluate the consistency of materials will also be very important. In this respect, the two compounds discovered through this study can be utilized as important markers for the effective quality control of various natural fermented products using this fungus in the future. In conclusion, it was found through this study that the extract of ginseng fermented with *Aspergillus cristatus* has high value as an active ingredient in effective skin anti-aging products. And isodihydroauroglaucin and flavoglaucin, which are related to these activities, can be utilized as effective quality standard markers for the development of excellent cosmeceuticals and pharmaceuticals.

## Data Availability

The raw data supporting the conclusions of this article will be made available by the authors, upon request.
