# Supplementary material for: Anti-aging efficacy of solid-state fermented ginseng with Aspergillus cristatus and its active metabolites
Source: Front Mol Biosci. 2022 Sep 29;9:984307. doi: 10.3389/fmolb.2022.984307 (PMC9556955; doi:10.3389/fmolb.2022.984307)
Supplement: Supplementary file 1 [file Table1.DOCX]

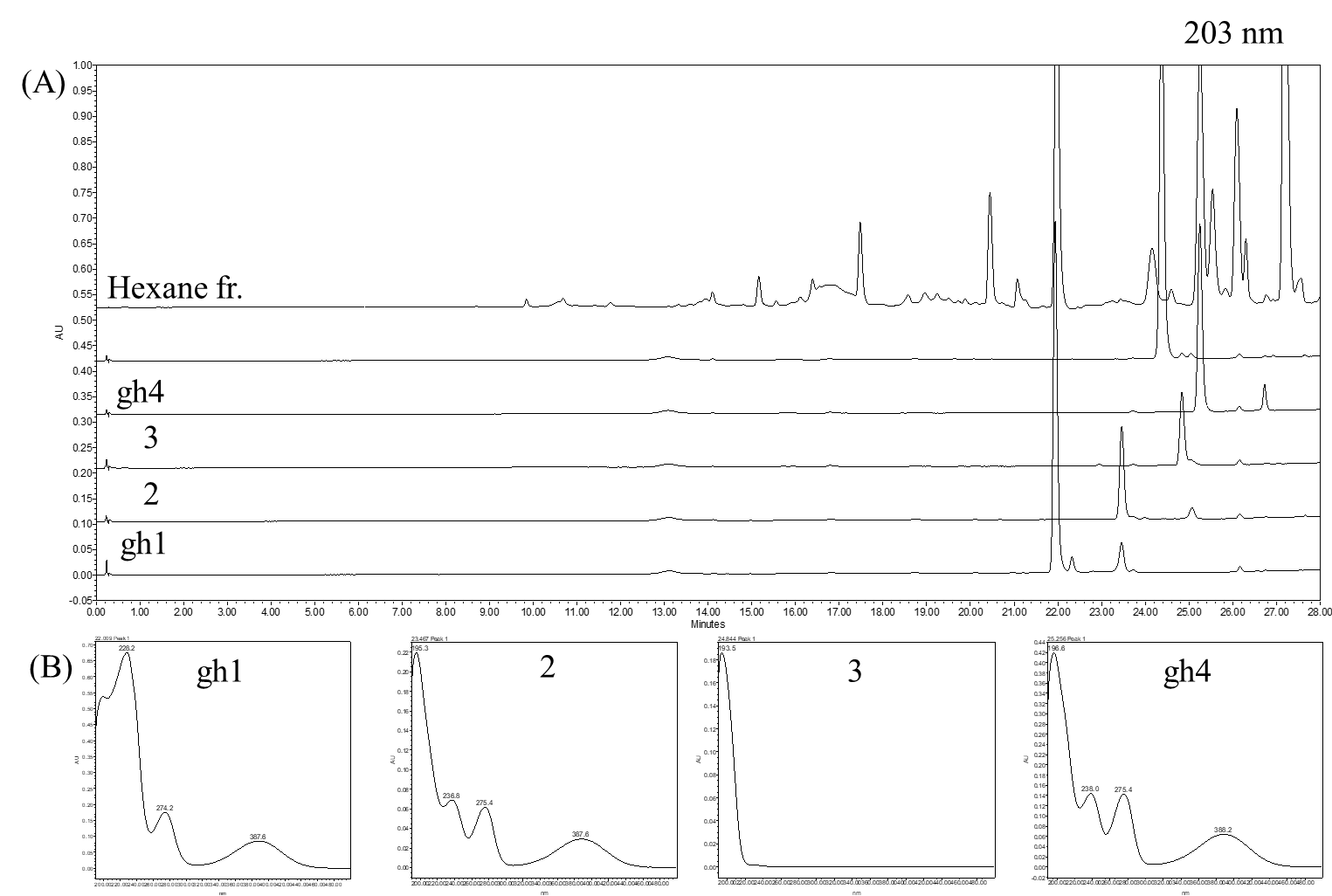


Figure S1. UHPLC chromatograms of hexane fraction of GFFG extract, gh1 and gh4. Gh1 (isodihydroauroglaucin) and gh4 (flavoglaucin) peaks were isolated from hexane fraction of GFFG extract (A). UV/Vis spectrum of gh1 and gh4 (B).


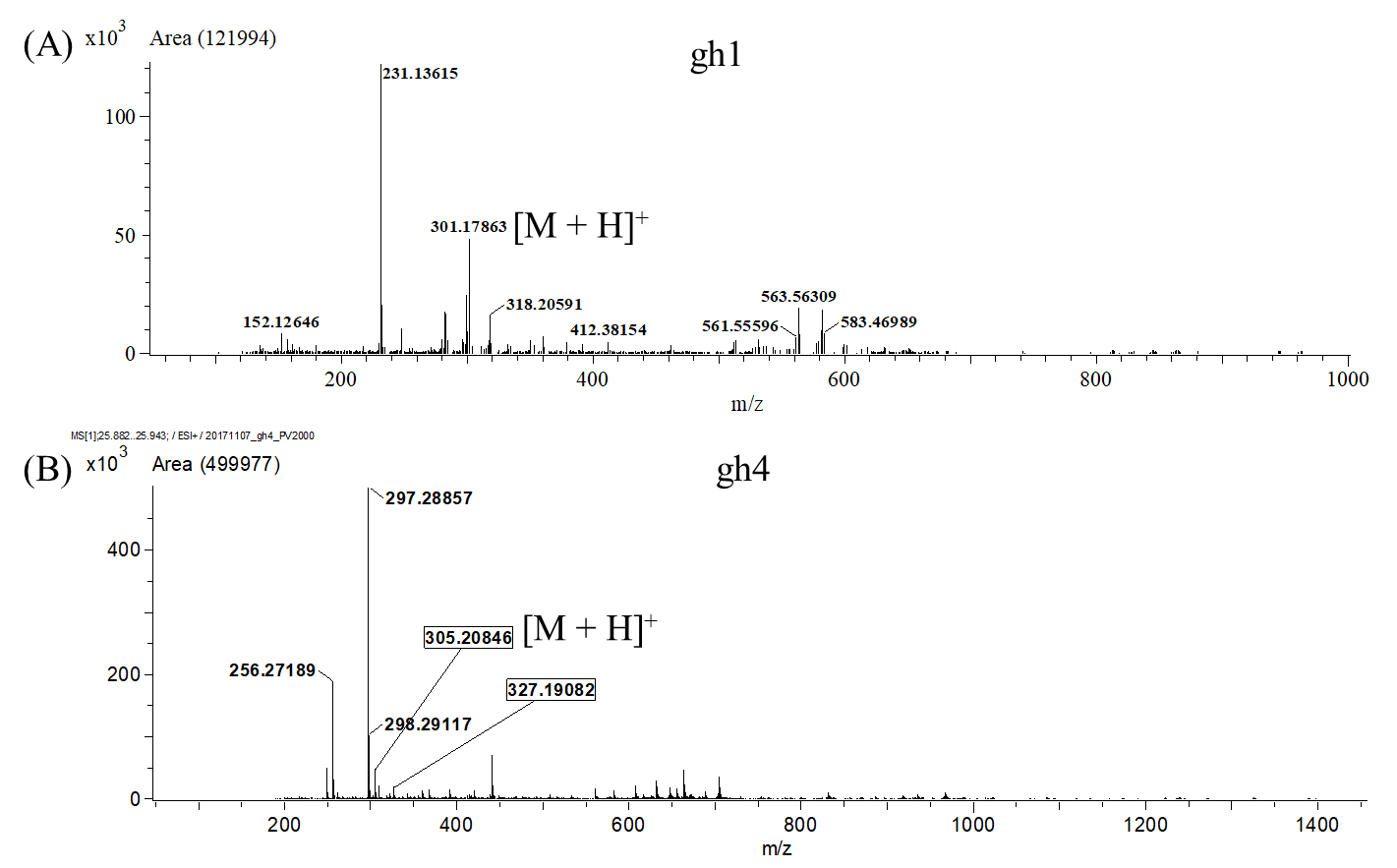


Figure S2. Mass spectrum of gh1 (A) and gh4 (B).


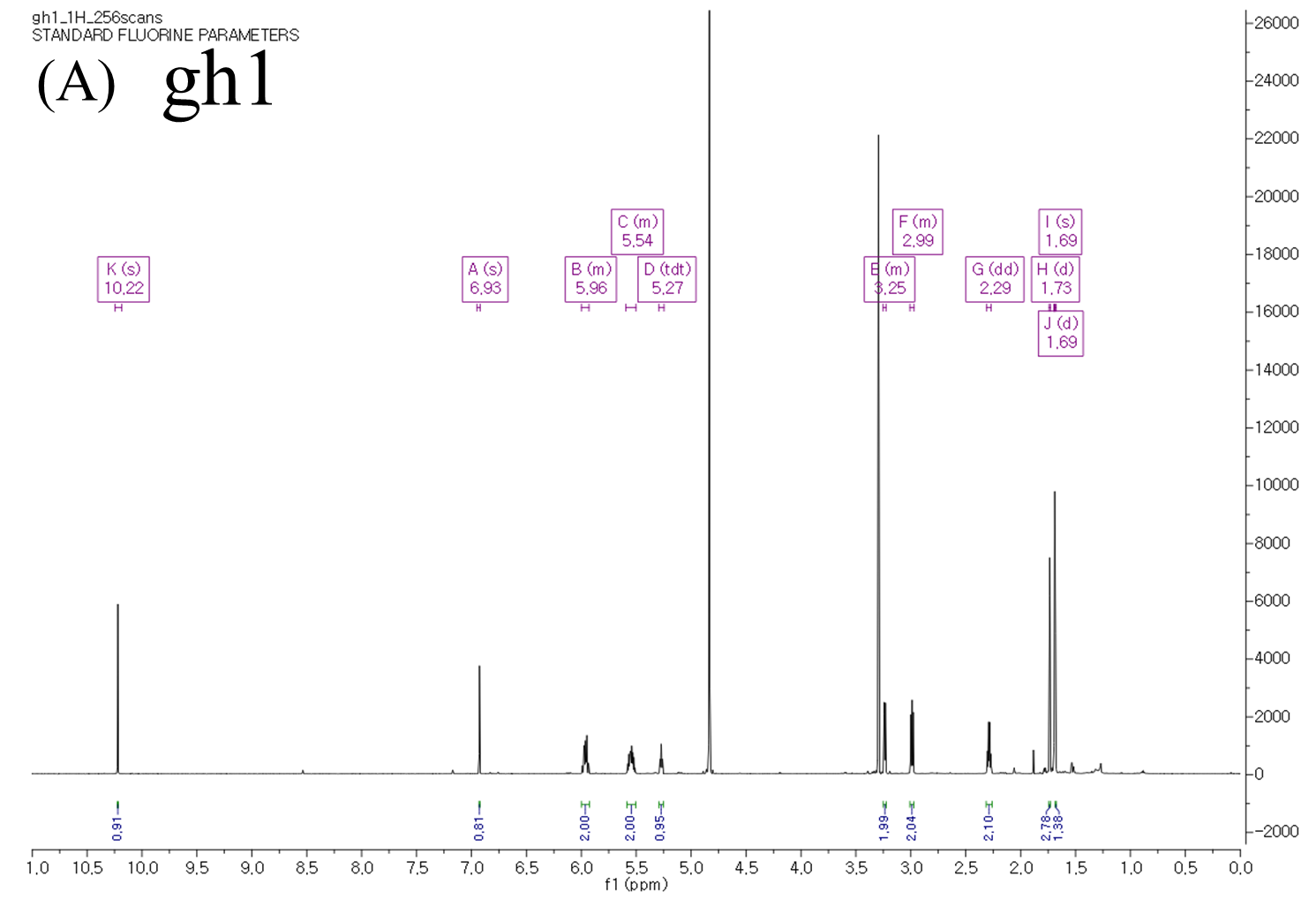


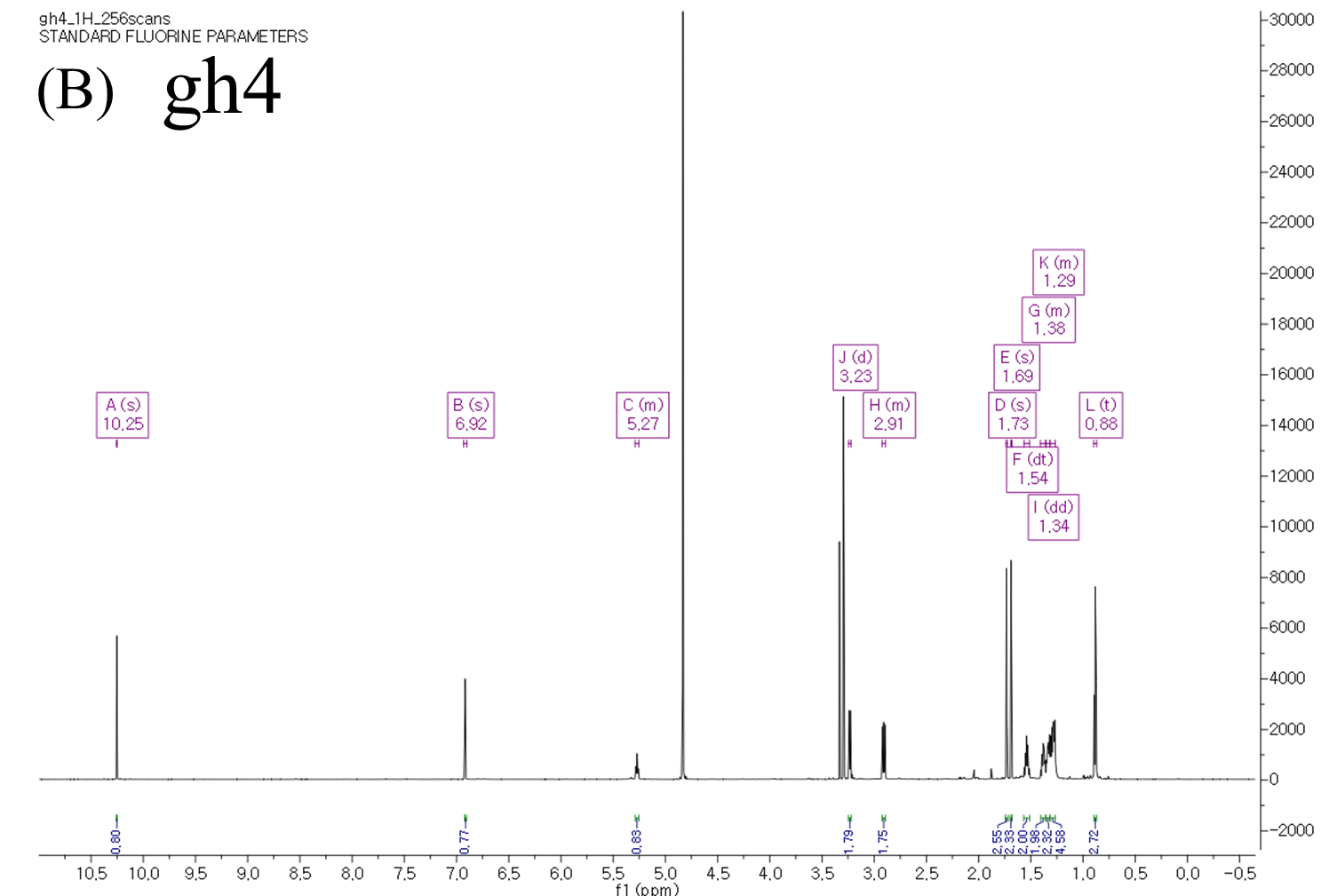


Figure S3. ^1^H-NMR spectrum of gh1 (A) and gh4 (B).

Table S1. ^1^H-NMR data comparison of compound gh1 and gh4 with the data previously reported.

| No. | gh1 | isodihydroauroglaucin | gh4 | flavoglaucin |
| --- | --- | --- | --- | --- |
| 1 | 1.7 (3H) | 1.70 (3H, s, H-4") | 0.88 (3H) | 0.88 (3H, t, J = 7.0 Hz, H-7') |
| 2 | 1.7 (3H) | 1.73 (3H, d, J = 6.7 Hz, H-7') | 1.29 (4H) | 1.28 (4H, m, H-5' and H-6') |
| 3 | 1.75 (3H) | 1.76 (3H, d, J = 1.0 Hz, H-5") | 1.34 (2H) | 1.30 (2H, m, H-4') |
| 4 | 2.3 (2H) | 2.34 (2H, q, J = 7.5 Hz, H-2') | 1.38 (2H) | 1.40 (2H, quintet, J = 7.6 Hz, H-3') |
| 5 | 3 (2H) | 2.98 (2H, t, J = 7.5 Hz, H-1') | 1.54 (2H) | 1.58 (2H, quintet, J = 7.6 Hz, H-2') |
| 6 | 3.25 (2H) | 3.29 (2H, d, J = 7.3 Hz, H-1") | 1.69 (3H) | 1.70 (3H, s, H-4") |
| 7 | 5.28 (H) | 5.28 (1H, m, H-2") | 1.73 (3H) | 1.75 (3H, s, H-5") |
| 8 | 5.55 (H) | 5.58 (1H, m, H-6') | 2.91 (2H) | 2.88 (2H, t, J = 7.6 Hz, H-1') |
| 9 | 5.57 (H) | 5.59 (1H, m, H-5') | 3.23 (2H) | 3.29 (2H, d, J = 7.3 Hz, H-1") |
| 10 | 5.96 (H) | 6.00 (1H, m, H-4') | 5.28 (1H) | 5.28 (1H, m, H-2") |
| 11 | 5.98 (H) | 6.02 (1H, m, H-3') | 6.92 (1H) | 6.89 (1H, s, H-4) |
| 12 | 6.94 (H) | 6.90 (1H, s, H-4) | 10.25 (1H) | 10.25 (1H, s, 1-CHO) |
| 13 | 10.23 (H) | 10.23 (1H, s, 1-CHO) |  |  |

Reference

Miyake, Y., et al., *Antioxidants produced by Eurotium herbariorum of filamentous fungi used for the manufacture of karebushi, dried bonito (Katsuobushi).* Biosci Biotechnol Biochem, 2009. **73**(6): p. 1323-7.
